# Supplementary material for: A Likelihood Approach for Real-Time Calibration of Stochastic Compartmental Epidemic Models
Source: PLoS Comput Biol. 2017 Jan 17;13(1):e1005257. doi: 10.1371/journal.pcbi.1005257 (PMC5240920; doi:10.1371/journal.pcbi.1005257)
Supplement: S1 File — (TAR.GZ) [file pcbi.1005257.s014.tar.gz › HSPH_Online-SI-Revision/PF08/n100-extreme/n100-extreme_PF_table4.pdf]

1	R0	{2.76052, 16.3913}	{2.17814, 16.592}	{{2.17243, 16.5183}, {2.18339, 16.6383}}	{21.0966, 1.22423}	inf	11577.	14751.5	{14662.7, 14833.5}	27.4207	0.105234	newly	4336.	5740.07	{5643., 5826.}	32.3817	0.121797	Reff	0.335762	0.63002	{0.627089, 0.63355}	87.6388	ar	92090.	87682.5	{87418., 87932.}	4.78609
2	R0	{2.18202, 15.1734}	{2.20056, 20.}	{{2.19247, 20.}, {2.20653, 20.}}	{0.849577, 31.8096}	inf	9523.	14858.1	{14795.5, 14926.4}	56.0232	0.193187	newly	3796.	4808.28	{4726., 4885.}	26.667	0.102622	Reff	0.491915	0.571137	{0.568059, 0.573696}	16.105	ar	84264.	95052.1	{94529., 95845.}	12.8027
3	R0	{2.57891, 12.5971}	{2.95915, 20.}	{{2.93389, 20.}, {2.97303, 20.}}	{14.7442, 58.7667}	inf	6810.	16304.8	{16280.1, 16312.4}	139.424	0.379166	newly	3181.	5084.05	{4997., 5170.}	59.8255	0.203613	Reff	0.320791	0.334227	{0.331458, 0.338303}	4.18868	ar	90302.	98573.3	{98350., 98842.}	9.15954
4	R0	{2.5856, 9.28508}	{3., 20.}	{{3., 20.}, {3., 20.}}	{16.0272, 115.399}	inf	4198.	22071.3	{21975.3, 22143.}	425.758	0.720785	newly	2428.	6919.4	{6816., 6994.}	184.984	0.454794	Reff	0.287622	0.388139	{0.386079, 0.390238}	34.9477	ar	90312.	103568.	{103236., 103924.}	14.6781
5	R0	{2.4406, 12.1341}	{2.85512, 20.}	{{2.84856, 20.}, {2.86378, 20.}}	{16.9844, 64.8248}	inf	8911.	20171.9	{20119.2, 20372.5}	126.37	0.354816	newly	4440.	6385.87	{6304., 6467.}	43.8259	0.15781	Reff	0.386079	0.436499	{0.435362, 0.438989}	13.0595	ar	88597.	97691.9	{97523., 97944.}	10.2655
6	R0	{2.72155, 15.4094}	{2.17945, 16.7385}	{{2.17672, 16.7103}, {2.1815, 16.7806}}	{19.9187, 8.62539}	inf	11194.	15882.3	{15842.7, 15930.6}	41.8826	0.151928	newly	4357.	6157.72	{6071., 6250.}	41.3294	0.150209	Reff	0.34629	0.668268	{0.666599, 0.670086}	92.9793	ar	91682.	88831.2	{88637., 89044.}	3.10941
7	R0	{2.63781, 14.3935}	{2.13033, 20.}	{{2.12341, 20.}, {2.1339, 20.}}	{19.2388, 38.9516}	inf	9306.	18063.1	{17980.9, 18120.4}	94.1021	0.288028	newly	3953.	6098.3	{5999., 6180.}	54.2702	0.188254	Reff	0.327906	0.826368	{0.823849, 0.830591}	152.013	ar	91192.	89565.2	{89262., 89823.}	1.78397
8	R0	{2.82391, 16.104}	{2.1439, 16.6764}	{{2.14212, 16.645}, {2.14659, 16.7228}}	{24.0806, 3.55432}	inf	11648.	15416.1	{15364., 15467.8}	32.3502	0.121723	newly	4404.	6017.52	{5924., 6098.}	36.6376	0.135545	Reff	0.322067	0.678012	{0.676103, 0.679861}	110.519	ar	92664.	87313.	{87047., 87587.}	5.77465
9	R0	{2.80645, 14.6457}	{2.1284, 17.2996}	{{2.12729, 17.2772}, {2.12921, 17.327}}	{24.1606, 18.1206}	inf	11375.	16931.	{16911.3, 16954.4}	48.844	0.172731	newly	4649.	6484.65	{6394., 6555.}	39.4848	0.144506	Reff	0.325885	0.763251	{0.761817, 0.764759}	134.209	ar	92567.	88504.3	{88255., 88743.}	4.38893
10	R0	{1.86213, 5.4271}	{3., 20.}	{{3., 20.}, {3., 20.}}	{61.1058, 268.521}	inf	1169.	20339.7	{20268.6, 20398.}	1639.92	1.24053	newly	1101.	6355.7	{6271., 6447.}	477.266	0.761353	Reff	0.480541	0.357885	{0.356225, 0.360008}	25.5247	ar	75362.	103050.	{102708., 103438.}	36.7403
11	R0	{2.27349, 14.6338}	{2.22466, 17.7446}	{{2.21839, 17.6447}, {2.22994, 17.845}}	{2.1476, 21.2573}	inf	9305.	13597.7	{13468., 13751.1}	46.1334	0.164737	newly	3974.	4894.37	{4822., 4965.}	23.1598	0.0904368	Reff	0.44615	0.541157	{0.536852, 0.545729}	21.2949	ar	85805.	92610.1	{92276., 92890.}	7.93092
12	R0	{2.77261, 14.1894}	{2.22165, 20.}	{{2.21478, 20.}, {2.22781, 20.}}	{19.8715, 40.9503}	inf	10089.	19475.6	{19423.2, 19513.7}	93.0381	0.285642	newly	4154.	6554.75	{6452., 6634.}	57.7937	0.198063	Reff	0.306207	0.816425	{0.814212, 0.818787}	166.625	ar	92508.	91364.2	{91065., 91742.}	1.23647
13	R0	{2.65402, 19.3935}	{2.19661, 20.}	{{2.19332, 20.}, {2.19907, 20.}}	{17.2347, 3.12734}	inf	15461.	15745.2	{15693.2, 15780.1}	1.83822	0.00790957	newly	4998.	5268.1	{5186., 5342.}	5.40416	0.0228277	Reff	0.43088	0.767627	{0.765365, 0.770564}	78.1533	ar	91184.	91676.2	{91339., 91968.}	0.547344
14	R0	{1.75297, 4.45354}	{2.18416, 17.8846}	{{2.18078, 17.8063}, {2.18746, 17.9734}}	{24.5978, 301.581}	inf	1073.	19715.6	{19635.4, 19778.2}	1737.43	1.26421	newly	1153.	7668.36	{7566., 7775.}	565.079	0.822848	Reff	0.522297	1.02704	{1.02193, 1.03253}	96.6389	ar	71263.	90521.4	{90286., 90745.}	27.0244
15	R0	{1.99928, 7.1501}	{2.90909, 20.}	{{2.90301, 20.}, {2.9177, 20.}}	{45.5068, 179.716}	inf	2594.	19499.8	{19401.4, 19694.8}	651.728	0.876051	newly	1932.	6137.79	{6050., 6236.}	217.691	0.501972	Reff	0.446419	0.409978	{0.404309, 0.413002}	8.1631	ar	79602.	99646.6	{98884., 100082.}	25.181
16	R0	{2.67398, 10.3134}	{2.94235, 20.}	{{2.94033, 20.}, {2.94504, 20.}}	{10.0363, 93.9225}	inf	4744.	21700.2	{21663.6, 21719.3}	357.424	0.660319	newly	2584.	6852.5	{6766., 6927.}	165.19	0.423539	Reff	0.275794	0.430055	{0.428573, 0.432976}	55.9332	ar	91289.	100135.	{99814., 100373.}	9.69009
17	R0	{2.44634, 16.3226}	{2.21375, 20.}	{{2.20872, 20.}, {2.21656, 20.}}	{9.50768, 22.5295}	inf	11754.	16283.8	{16245.7, 16322.8}	38.5383	0.141568	newly	4470.	5314.55	{5236., 5394.}	18.8937	0.0751267	Reff	0.431632	0.60921	{0.606241, 0.613987}	41.1409	ar	88710.	94469.1	{94122., 94814.}	6.49208
18	R0	{2.02168, 10.1676}	{2.11223, 20.}	{{2.1094, 20.}, {2.11404, 20.}}	{4.47892, 96.7033}	inf	6854.	15233.1	{15186.6, 15267.8}	122.252	0.346841	newly	4020.	5253.81	{5152., 5340.}	30.6918	0.116212	Reff	0.515609	0.948808	{0.947383, 0.950287}	84.0169	ar	80299.	87348.7	{87003., 87667.}	8.77931
19	R0	{2.89969, 15.009}	{2.23109, 20.}	{{2.22691, 20.}, {2.23388, 20.}}	{23.0578, 33.2534}	inf	11247.	19760.2	{19685.9, 19816.6}	75.693	0.244753	newly	4512.	6645.66	{6556., 6728.}	47.2886	0.168141	Reff	0.292405	0.818783	{0.816488, 0.822053}	180.017	ar	93349.	91785.	{91467., 92113.}	1.67542
20	R0	{2.68933, 7.34928}	{3., 20.}	{{3., 20.}, {3., 20.}}	{11.5519, 172.136}	inf	2277.	27511.7	{27462.4, 27554.6}	1108.24	1.08215	newly	1542.	8858.91	{8760., 8952.}	474.508	0.75928	Reff	0.246316	0.561532	{0.559908, 0.56344}	127.972	ar	91574.	100493.	{100207., 100751.}	9.74016
21	R0	{2.15352, 9.28074}	{2.94456, 20.}	{{2.94115, 20.}, {2.94723, 20.}}	{36.7324, 115.5}	inf	5169.	19392.3	{19369.1, 19405.5}	275.166	0.574223	newly	3172.	6099.93	{6017., 6184.}	92.3055	0.283966	Reff	0.421315	0.400487	{0.39949, 0.402124}	4.94341	ar	83845.	99529.3	{99386., 99654.}	18.7063
22	R0	{2.21953, 9.04387}	{3., 20.}	{{3., 20.}, {3., 20.}}	{35.1638, 121.144}	inf	4637.	19151.5	{19065.7, 19282.1}	313.014	0.61596	newly	2827.	5958.1	{5870., 6049.}	110.757	0.323758	Reff	0.397451	0.334409	{0.332736, 0.336209}	15.8616	ar	84856.	102297.	{101971., 102556.}	20.5534
23	R0	{2.21987, 14.5347}	{1.95176, 20.}	{{1.95025, 20.}, {1.95291, 20.}}	{12.0778, 37.6017}	inf	9287.	14752.	{14731., 14771.4}	58.8461	0.200976	newly	3912.	5014.99	{4928., 5081.}	28.195	0.107842	Reff	0.465951	0.860358	{0.859045, 0.861824}	84.6457	ar	85067.	83926.	{83590., 84201.}	1.34127
24	R0	{2.84268, 17.9659}	{1.95473, 20.}	{{1.95308, 20.}, {1.95624, 20.}}	{31.2363, 11.322}	inf	14057.	14854.9	{14809.5, 14890.9}	5.67647	0.023977	newly	4888.	5067.81	{4973., 5154.}	3.6876	0.0156926	Reff	0.351242	0.890995	{0.889221, 0.893409}	153.67	ar	92836.	83731.7	{83357., 84062.}	9.80691
25	R0	{2.52706, 10.3679}	{2.10722, 20.}	{{2.10092, 20.}, {2.11013, 20.}}	{16.614, 92.9031}	inf	5201.	18196.3	{18092., 18270.}	249.862	0.543894	newly	2743.	6281.24	{6204., 6363.}	128.992	0.359794	Reff	0.316085	0.967909	{0.965917, 0.971278}	206.218	ar	89623.	87979.3	{87666., 88281.}	1.83398
26	R0	{2.62835, 19.1088}	{2.27871, 20.}	{{2.27332, 20.}, {2.2832, 20.}}	{13.3025, 4.66382}	inf	14411.	16002.8	{15951.5, 16041.4}	11.0457	0.0455002	newly	4701.	5168.13	{5085., 5250.}	9.93682	0.0411092	Reff	0.418354	0.558282	{0.555755, 0.561614}	33.447	ar	90908.	96326.1	{96034., 96606.}	5.95994
27	R0	{2.76831, 19.4155}	{2.15569, 16.9845}	{{2.14726, 16.8782}, {2.15855, 17.0522}}	{22.1297, 12.5211}	inf	12467.	12180.9	{12051.8, 12283.1}	2.78022	0.0122129	newly	3937.	4569.06	{4487., 4649.}	16.0544	0.0646151	Reff	0.347063	0.551857	{0.548045, 0.556931}	59.0077	ar	92175.	89378.	{89110., 89601.}	3.03445
28	R0	{2.96609, 16.8903}	{3., 20.}	{{3., 20.}, {3., 20.}}	{1.14326, 18.4112}	inf	11510.	15801.1	{15653.4, 15979.2}	37.2813	0.137599	newly	4186.	4897.98	{4816., 4993.}	17.0086	0.0681694	Reff	0.274037	0.305738	{0.303338, 0.308993}	11.5683	ar	94024.	100466.	{100074., 100817.}	6.85194
29	R0	{2.17046, 14.39}	{1.98183, 20.}	{{1.97861, 20.}, {1.98418, 20.}}	{8.6906, 38.9854}	inf	10054.	15223.6	{15182.7, 15254.1}	51.4184	0.180178	newly	4398.	5163.89	{5077., 5243.}	17.4145	0.0696931	Reff	0.504914	0.855803	{0.853705, 0.85856}	69.4947	ar	84001.	85436.7	{85155., 85703.}	1.70919
30	R0	{2.88772, 12.4647}	{3., 20.}	{{3., 20.}, {3., 20.}}	{3.88819, 60.4531}	inf	8175.	21926.5	{21889.5, 21959.3}	168.215	0.428482	newly	3775.	6886.39	{6789., 6976.}	82.4209	0.261053	Reff	0.263591	0.399214	{0.398033, 0.400648}	51.4519	ar	93273.	101524.	{101222., 101776.}	8.8462
31	R0	{2.46831, 8.14308}	{3., 20.}	{{3., 20.}, {3., 20.}}	{21.5406, 145.607}	inf	3768.	25052.4	{24957., 25127.5}	564.873	0.822737	newly	2494.	7931.63	{7836., 8021.}	218.028	0.502445	Reff	0.317474	0.454362	{0.452619, 0.456487}	43.1179	ar	88762.	103343.	{102828., 103776.}	16.4269
32	R0	{1.93236, 7.38987}	{3., 20.}	{{3., 20.}, {3., 20.}}	{55.2506, 170.641}	inf	3204.	18356.5	{18302.9, 18542.9}	472.926	0.758094	newly	2353.	5719.5	{5624., 5798.}	143.073	0.385708	Reff	0.482588	0.340937	{0.339222, 0.343548}	29.3523	ar	77654.	100850.	{100264., 101123.}	29.8713
33	R0	{2.82251, 17.6954}	{2.89269, 20.}	{{2.86334, 20.}, {2.92464, 20.}}	{2.48634, 13.0237}	inf	15003.	17953.4	{17695.3, 18298.7}	19.6656	0.0779327	newly	5209.	5630.83	{5504., 5752.}	8.0981	0.033757	Reff	0.365938	0.379579	{0.367148, 0.391549}	3.78739	ar	92745.	98091.1	{97636., 98444.}	5.76429
34	R0	{1.87083, 12.3994}	{2.25973, 20.}	{{2.24401, 20.}, {2.27055, 20.}}	{20.7875, 61.2981}	inf	8229.	14996.7	{14903.8, 15093.1}	82.2421	0.260643	newly	4286.	4928.29	{4829., 5013.}	14.9858	0.0606047	Reff	0.630488	0.655261	{0.647266, 0.663501}	3.92907	ar	76282.	94466.4	{93830., 95079.}	23.8383
35	R0	{2.95779, 16.3003}	{2.17281, 16.2346}	{{2.17013, 16.216}, {2.17449, 16.2619}}	{26.5393, 0.40334}	inf	14081.	16572.4	{16549.1, 16596.1}	17.6937	0.0707528	newly	5272.	6678.82	{6598., 6770.}	26.6847	0.102705	Reff	0.314472	0.715673	{0.713691, 0.717656}	127.579	ar	93873.	87937.8	{87735., 88148.}	6.32258
36	R0	{2.18718, 14.261}	{2.17735, 16.1731}	{{2.1678, 16.089}, {2.18402, 16.2524}}	{0.450577, 13.4077}	inf	9571.	12169.8	{12003.8, 12349.1}	27.1532	0.104301	newly	4189.	4760.18	{4670., 4855.}	13.6352	0.055466	Reff	0.488835	0.542116	{0.536496, 0.549476}	10.8997	ar	84379.	88503.8	{88190., 88745.}	4.88843
37	R0	{2.44912, 9.31118}	{3., 20.}	{{3., 20.}, {3., 20.}}	{22.493, 114.796}	inf	4364.	20402.	{20356.2, 20440.9}	367.508	0.669788	newly	2540.	6386.52	{6292., 6481.}	151.438	0.400408	Reff	0.332493	0.373741	{0.372189, 0.375125}	12.4058	ar	88442.	102160.	{101810., 102477.}	15.5104
38	R0	{2.05949, 5.72514}	{3., 20.}	{{3., 20.}, {3., 20.}}	{45.6671, 249.336}	inf	1447.	25668.3	{25618., 25714.7}	1673.9	1.24893	newly	1229.	8183.94	{8054., 8308.}	565.902	0.823383	Reff	0.397749	0.502432	{0.500897, 0.504159}	26.3187	ar	81587.	100750.	{100471., 101020.}	23.4879
39	R0	{1.75505, 8.52404}	{2.33948, 20.}	{{2.3189, 20.}, {2.34988, 20.}}	{33.2996, 134.631}	inf	4589.	18027.6	{17918.1, 18116.4}	292.843	0.594215	newly	3152.	5842.36	{5741., 5931.}	85.3541	0.267973	Reff	0.605615	0.583795	{0.577786, 0.590734}	3.60292	ar	70925.	97372.7	{96925., 97938.}	37.2897
40	R0	{2.24724, 13.7861}	{2.30118, 17.2948}	{{2.27215, 17.0629}, {2.32692, 17.4944}}	{2.4045, 25.451}	inf	8230.	13638.4	{13353.8, 13910.1}	65.7153	0.219313	newly	3630.	4951.77	{4866., 5038.}	36.4124	0.13481	Reff	0.451088	0.462644	{0.452055, 0.473292}	2.75819	ar	85257.	96673.8	{95796., 97406.}	13.391
41	R0	{1.77635, 8.62406}	{2.21061, 15.128}	{{2.11423, 14.2372}, {2.2676, 15.8084}}	{24.4466, 75.4158}	inf	4190.	11896.5	{10715., 12643.9}	183.925	0.450381	newly	2830.	4914.99	{4696., 5159.}	73.6746	0.239228	Reff	0.557952	0.530564	{0.505577, 0.556633}	5.11532	ar	72941.	96147.7	{94195., 97850.}	31.8157
42	R0	{1.80397, 5.4932}	{3., 20.}	{{3., 20.}, {3., 20.}}	{66.2999, 264.087}	inf	1509.	20373.5	{20333.4, 20410.2}	1250.13	1.13038	newly	1451.	6372.	{6283., 6463.}	339.145	0.642583	Reff	0.511444	0.365606	{0.364012, 0.367709}	28.5149	ar	73232.	101406.	{101071., 101705.}	38.4724
43	R0	{2.83096, 16.7077}	{1.90925, 20.}	{{1.90794, 20.}, {1.90954, 20.}}	{32.5584, 19.7053}	inf	13176.	14266.2	{14236., 14276.8}	8.27403	0.0345232	newly	4750.	5010.04	{4934., 5080.}	5.47453	0.023113	Reff	0.342093	1.04954	{1.0487, 1.05122}	206.8	ar	92740.	82207.9	{81885., 82481.}	11.3565
44	R0	{2.73678, 11.6021}	{3., 20.}	{{3., 20.}, {3., 20.}}	{9.61787, 72.3826}	inf	5400.	18129.	{18099.1, 18154.8}	235.722	0.525979	newly	2650.	5652.63	{5549., 5739.}	113.307	0.328976	Reff	0.263771	0.339287	{0.337851, 0.340733}	28.6296	ar	92003.	100298.	{100081., 100520.}	9.01585
45	R0	{2.14235, 6.98601}	{2.94287, 20.}	{{2.93852, 20.}, {2.94581, 20.}}	{37.3663, 186.286}	inf	1905.	21480.4	{21460.6, 21499.2}	1027.58	1.05215	newly	1420.	6780.52	{6690., 6864.}	377.501	0.678954	Reff	0.389458	0.428008	{0.42644, 0.430645}	9.89851	ar	82974.	99525.4	{99308., 99704.}	19.9477
46	R0	{2.23409, 9.21289}	{2.8483, 20.}	{{2.83969, 20.}, {2.85129, 20.}}	{27.4926, 117.087}	inf	5803.	22076.7	{22045.7, 22095.5}	280.437	0.580282	newly	3539.	7021.65	{6925., 7107.}	98.4077	0.297535	Reff	0.407766	0.477397	{0.47556, 0.482061}	17.0763	ar	85163.	99322.2	{99064., 99522.}	16.626
47	R0	{2.12258, 8.15265}	{3., 20.}	{{3., 20.}, {3., 20.}}	{41.3374, 145.319}	inf	3887.	20211.1	{20110.6, 20304.}	419.965	0.715971	newly	2681.	6315.42	{6221., 6411.}	135.562	0.372078	Reff	0.420441	0.363752	{0.361196, 0.36603}	13.4831	ar	82690.	104572.	{103998., 105128.}	26.4624
48	R0	{2.71771, 10.4636}	{3., 20.}	{{3., 20.}, {3., 20.}}	{10.3871, 91.1388}	inf	6665.	23743.1	{23703.7, 23784.3}	256.235	0.551737	newly	3585.	7482.55	{7371., 7574.}	108.718	0.319537	Reff	0.28718	0.419223	{0.417552, 0.421008}	45.9788	ar	91716.	102032.	{101617., 102359.}	11.2473
49	R0	{2.44369, 6.66477}	{3., 20.}	{{3., 20.}, {3., 20.}}	{22.7652, 200.085}	inf	1262.	25419.7	{25323.5, 25510.5}	1914.24	1.30411	newly	921.	8071.94	{7987., 8160.}	776.432	0.942699	Reff	0.285399	0.466185	{0.463638, 0.468362}	63.3451	ar	88827.	103440.	{102979., 103898.}	16.4508
50	R0	{2.77383, 17.9775}	{2.16332, 20.}	{{2.16061, 20.}, {2.16485, 20.}}	{22.0096, 11.2502}	inf	11601.	16296.	{16250.9, 16319.6}	40.4705	0.147585	newly	3992.	5350.13	{5273., 5431.}	34.0213	0.127147	Reff	0.332083	0.661203	{0.659453, 0.663482}	99.1077	ar	92334.	91618.8	{91309., 91877.}	0.774568
